# Measuring Symmetry, Asymmetry and Randomness in Neural Network Connectivity

**DOI:** 10.1371/journal.pone.0100805

**Published:** 2014-07-09

**Authors:** Umberto Esposito, Michele Giugliano, Mark van Rossum, Eleni Vasilaki

**Affiliations:** 1 Department of Computer Science, University of Sheffield, Sheffield, United Kingdom; 2 Theoretical Neurobiology and Neuroengineering Laboratory, Department of Biomedical Sciences, University of Antwerp, Wilrijk, Belgium; 3 Laboratory of Neural Microcircuitry, Brain Mind Institute, École polytechnique fédérale de Lausanne, Lausanne, Switzerland; 4 School of Informatics, University of Edinburgh, Edinburgh, United Kingdom; University of Genova, Italy

## Abstract

Cognitive functions are stored in the connectome, the wiring diagram of the brain, which exhibits non-random features, so-called *motifs*. In this work, we focus on bidirectional, symmetric motifs, i.e. two neurons that project to each other via connections of equal strength, and unidirectional, non-symmetric motifs, i.e. within a pair of neurons only one neuron projects to the other. We hypothesise that such motifs have been shaped via activity dependent synaptic plasticity processes. As a consequence, learning moves the distribution of the synaptic connections away from randomness. Our aim is to provide a global, macroscopic, single parameter characterisation of the statistical occurrence of bidirectional and unidirectional motifs. To this end we define a symmetry measure that does not require any *a priori* thresholding of the weights or knowledge of their maximal value. We calculate its mean and variance for random uniform or Gaussian distributions, which allows us to introduce a confidence measure of how significantly symmetric or asymmetric a specific configuration is, i.e. how likely it is that the configuration is the result of chance. We demonstrate the discriminatory power of our symmetry measure by inspecting the eigenvalues of different types of connectivity matrices. We show that a Gaussian weight distribution biases the connectivity motifs to more symmetric configurations than a uniform distribution and that introducing a random synaptic pruning, mimicking developmental regulation in synaptogenesis, biases the connectivity motifs to more asymmetric configurations, regardless of the distribution. We expect that our work will benefit the computational modelling community, by providing a systematic way to characterise symmetry and asymmetry in network structures. Further, our symmetry measure will be of use to electrophysiologists that investigate symmetry of network connectivity.

## Introduction

It is widely believed that cognitive functions are stored in the so-called *connectome* [1], [2], the wiring diagram of the brain. Due to improvements in technology, experimental techniques and computational paradigms [3], [4], the investigation of the connectome, known as *connectomics*, has generated great excitement [5] and has made significant progress [6]–[9] resulting in a rapid proliferation of neuroscience datasets [10]–[13].

Studies on the brain wiring diagram have shown that connectivity is non-random, highlighting the existence of specific connectivity motifs at the microcircuit level, see for instance [14]–[17]. Of particular interest are the motifs that exhibit bidirectional (reciprocal) and unidirectional (non-reciprocal) connections between pairs of neurons. More specifically, theoretical work [18] studied the development of unidirectional connectivity due to long-term plasticity in an artificial network of spiking neurons under a *temporal coding scheme*, where it is assumed that the time at which neurons fire carries out important information. This finding is correlated to unidirectional connectivity observed in somatosensory cortex, see [19]. In [18] the development of bidirectional connectivity in the same network under a *frequency coding scheme*, where information is transmitted in the firing rate of the neurons, was also studied and correlated to bidirectional connectivity found in the visual cortex [14]. Complementary to this work, in [20], [21] the authors explored the experimentally identified correlation of bidirectional and unidirectional connectivity to short-term synaptic dynamics, see [22], by studying the development of connectivity in networks with facilitating and depressing synapses due to the interaction of short-term and long-term plasticities. The role of synaptic long-term plasticity in structures formation within networks has been also investigated in [23]–[25].

Similar to [18] and [20], [21], we hypothesise that the above mentioned motifs have been shaped via activity dependent synaptic plasticity processes, and that learning moves the distribution of the synaptic connections away from randomness. Our aim is to provide a global, macroscopic, single parameter characterisation of the statistical occurrence of bidirectional and unidirectional motifs. To this end:

1. We define a symmetry measure that does not require any a priori thresholding of the weights or knowledge of their maximal value, and hence is applicable to both simulations and experimental data.
2. We calculate the mean and variance of this symmetry measure for random uniform or Gaussian distributions, which allows us to introduce a confidence measure of how significantly symmetric or asymmetric is a specific configuration, i.e. how likely it is that the configuration is the result of chance.
3. We demonstrate the discriminatory power of our symmetry measure by inspecting the eigenvalues of different types of connectivity matrices, given that symmetric matrices are known to have real eigenvalues.
4. We show that a Gaussian distribution biases the connectivity motifs to more symmetric configurations than a uniform distribution and that introducing a random synaptic pruning, mimicking developmental regulation in synaptogenesis, biases the connectivity motifs to more asymmetric configurations, regardless of the distribution. Our statistics of the symmetry measure allows us to correctly evaluate the significance of a symmetric or asymmetric network configuration in both these cases.
5. Our symmetry measure allows us to observe the evolution of a specific network configuration, as we exemplify in our results.

We expect that our work will benefit the computational modelling community, by providing a systematic way to characterise symmetry and asymmetry in network structures. Further, our symmetry measure will be of use to electrophysiologists that may investigate symmetric or asymmetric network connectivity.

## Methods

In what follows, we first define a novel measure that quantifies the degree of symmetry in a neuronal network with excitatory synaptic connections. More specifically, we describe the strength of the synaptic efficacies between the neurons by the elements of a square matrix, i.e. the connectivity matrix, to which we associate a number that quantifies the similarity of the elements above the matrix diagonal to those below the diagonal. We further study this measure from a statistical point of view, by means of both analytical tools and numerical simulations. Aiming to associate a significance value to the measure, i.e. the probability that a certain symmetric or non-symmetric configuration is the result of chance, we consider random synaptic efficacies drawn from uniform and Gaussian distributions. We also study how our symmetry measure is affected by the anatomical disconnection of neurons in a random manner, i.e. zeroing some entries in the connectivity matrix. Finally, we anticipate that connectivity distributions are modified by activity-dependent processes and we describe the structure of the network we use as a demonstrative example in the Results section.

### Definitions

Let us consider the adjacency (or connectivity) matrix 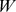 of a weighted directed network [26], composed of 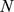 vertices and without self-edges. The 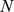 vertices represent the neurons, with 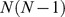 possible synaptic connections among them. The synaptic efficacy between two neurons is expressed as a positive element 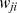 in the adjacency matrix. 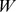 is thus composed by positive elements off-diagonal, taking values in the bounded range 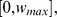 and by zero diagonal entries. We define 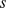 as a measure of the symmetry of 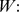


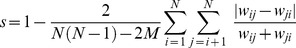
(1)where 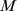 is the number of instances where both 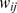 and 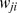 are zero, i.e. there is no connection between two neurons. The term 
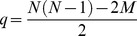
 is a normalisation factor that represents the total number of synaptic connection pairs that have at least one non-zero connection. A value of 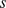 near 0 indicates that there are virtually no reciprocal connections in the network, while a value of 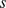 near 1 indicates that virtually all connections are reciprocal. We exclude (0,0) pairs from our definition of the symmetry measure. Mathematically such pairs would introduce undefined terms to Eq. (1). In addition, conceptually, we expect that small weights will not be experimentally measurable. It is then reasonable to exclude them, expecting to effectively increase the signal to noise ratio.

#### Pruning and Plasticity

We assume that a connection 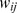 is permanently disconnected and set to 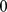 with probability 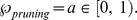 Consequently, the probability that two neurons 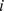 and 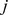 are mutually disconnected, i.e. 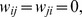 is 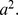 When a connection is permanently *pruned* in such a way, its efficacy remains 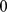 all the time, whereas the off-diagonal non-pruned values of the adjacency matrix 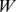 change slowly in time, as a result of activity-dependent synaptic plasticity. We consider that this procedure correlates with developmental mechanisms associated with or following synaptogenesis.

#### Unidirectional and Bidirectional connection pairs

We associate the quantity 
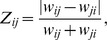
 i.e. the term of the summation in the Eq. (1) to the neuronal pair 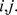 This term maps the strength of the connections between two neurons to a single variable. Each connection pair can therefore be bidirectional if 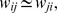 unidirectional if 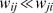 or 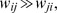 or none of the two. As a consequence, a network can be dominated by bidirectional connectivity, by unidirectional connectivity, or it may exhibit random features.

#### Weight Bounds

In what follows we consider the case of 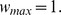 Due to the term 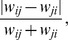 this can be done without loss of generality.

### Statistics of s

Let us consider a large number of 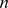 instances of a network whose connection weights are randomly distributed. Each adjacency matrix can be evaluated via our symmetry measure. We rewrite Eq. 1 as:
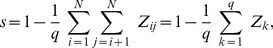
(2)where 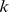 is a linear index running over all the 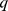 non-zero “connection pairs” within the network. We can then estimate the mean 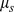 and variance 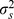 of 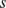 over all 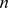 networks as:
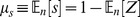
(3)

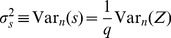
(4)where the notation 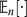 and 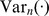 implies that the expected value and variance are computed along the 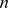 different representations of the network.

Eq. (3), (4) allow us to transfer the statistical analysis from 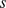 to 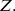 To derive theoretical formulas for mean value and variance of 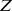 we use the fact that its probability density function (PDF), 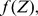 can be written as a joint distribution, 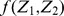 where we have introduced the notation 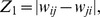 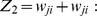


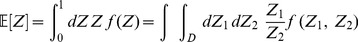
(5)within the range 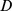 defined by 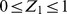 and 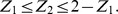 Similarly, we can calculate the variance as follows:
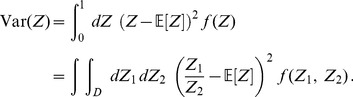
(6)


We note that mean value and variance of 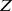 can be numerically estimated either by using a large set of small networks or on a single very large network: What matters is that the total number of connection pairs, given by the product 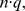 is sufficiently large to guarantee good statistics and that connection pairs are independent of each other. In the calculations below, we assume a very large adjacency matrix.

### Adjacency matrix with uniform random values

We first consider a network with randomly distributed connections without pruning, followed by the more general case where pruning is taken into account.

#### Fully connected network

**Figure 1:**
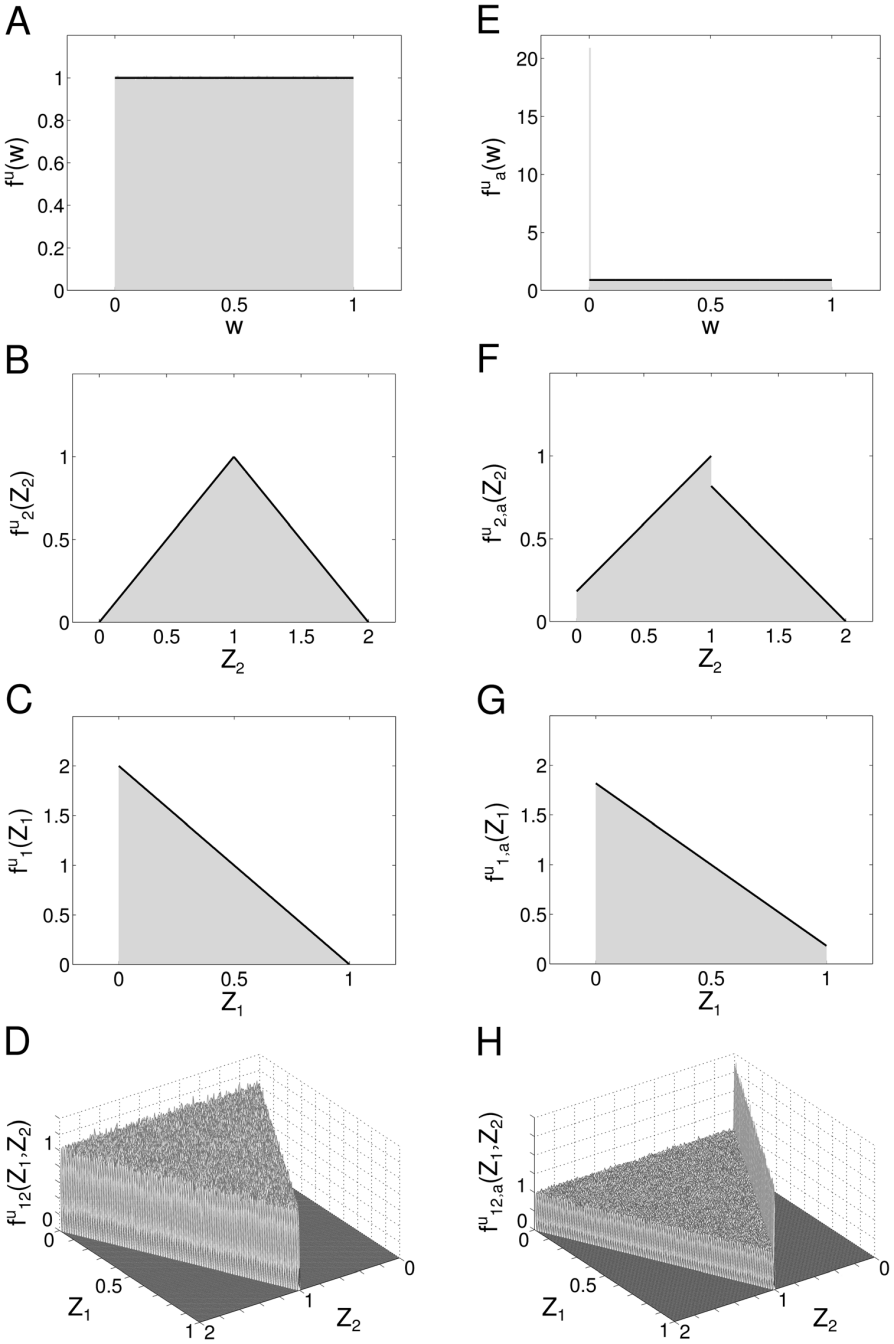
Probability density functions for the case of uniformly distributed connections. **A** Distribution of the uniform variable 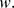 **B** Distribution of the sum 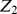 of two uniform variables. **C** Distribution of the absolute difference 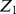 of two uniform variables. **D** Joint distribution of 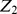 and 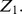 **E, F, G** The same as A, B and C but with pruning 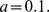 **H** The same as D but with pruning 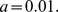 In all figures, *Grey shaded area*: histograms from simulations, *Black lines and surfaces*: theoretical results (see Eq. (7)–(13)).

For the uniform distribution 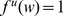 for 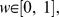 see Fig. 1A. The probability of having 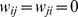 for at least one pair 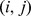 is negligible, hence 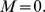 It is straightforward to derive the distributions 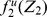 and 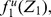 depicted in Fig. 1B,C correspondingly:
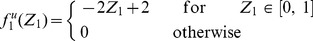
(7)

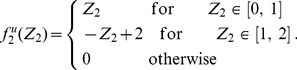
(8)


We can therefore obtain the joint PDF (Fig. 1D):
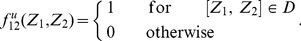
(9)


#### Pruning

Introducing pruning to the elements of the adjacency matrix, with probability 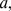 corresponds to a discontinuous probability distribution function of 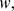 that can be written as a sum of a continuous function and of a Dirac's Delta centred in 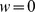 (see also Fig. 1E):
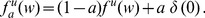
(10)


Now the (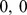) pairs have to be explicitly excluded from the distributions of 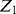 and 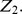 Also, the number of pairs of the type (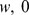) increases, resulting in the appearance of a uniform contribution in the region 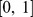 in both the PDF of 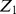 and 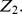 Their final exact profile can be obtained by considering the possible combinations of drawing 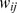 and 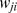 from the above pruned distribution and their corresponding probability of occurrence. There are four contributions: 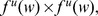 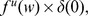 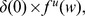 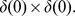 The last term, which describes the (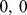) pairs, has to be subtracted and the remaining expression has to be renormalised. The results are graphically shown in Fig. 1F, 1G and are mathematically described by the following expressions:
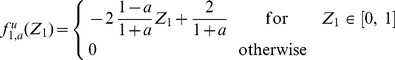
(11)

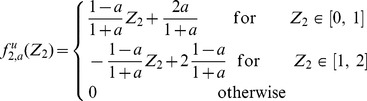
(12)


The joint PDF is a mixture of two uniform distributions: the unpruned distribution 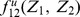 and the contribution from the pruning, 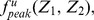 which is a delta peak along the line 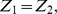 see Fig. 1H. To obtain 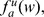 the two unitary distributions are mixed with some coefficients 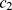 and 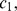 satisfying the normalisation condition 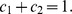 With the same arguments used for 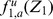 and 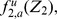 we can derive the relation between 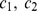 and 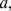 so that we can finally write:
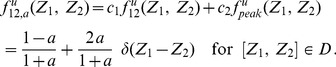
(13)


#### Expected value and variance of 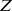


We can calculate mean value and variance of 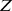 by plugging Eq. (13) into Eqs (5) and (6):
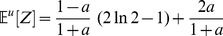
(14)

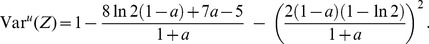
(15)


#### Expected value and variance of 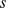


By combining the above results with Eqs (3) and (4), we can derive the final formulas for the expected value and variance of 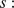


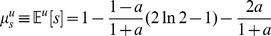
(16)

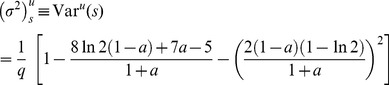
(17)


### Adjacency matrix with Gaussian-distributed random values

The procedure described above to derive the joint PDF of 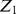 and 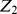 is applicable to any distribution. In what follows, we consider a network with initial connections drawn by a truncated Gaussian distribution.

#### Distribution of connections

Whereas the uniform distribution is well defined in any finite interval, the Gaussian distribution requires some considerations. Strictly speaking, any Gaussian distribution is defined over the entire real axes. For practical reasons, however, for any finite network 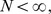 the maximum and the minimum values of the weights, 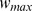 and 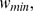 are always well defined, and therefore the actual distribution is a truncated Gaussian. To be able to consider the truncated Gaussian distribution as Gaussian with satisfactory accuracy, we require that the portion of the Gaussian enclosed in the region 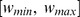 is as close as possible to 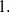 This means that the distribution has to be narrow enough with respect to the interval of definition 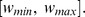 Also, by definition, the distribution has to be symmetric in 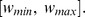 Because we are considering only excitatory connections then 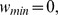 so as the mean value has to be 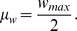 On the other hand, the narrowness imposes a condition on the standard deviation of the distribution: 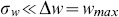. Since we can set 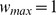 without loss of generalization, the entire study on all the possible Gaussian distributions can be limited to a special class, 
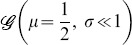
.

#### The choice of 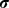


To guarantee a good approximation of a Gaussian distribution, we define the truncated Gaussian distribution such that points within 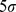 fall in [0, 1] leading to 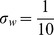 and a truncation error 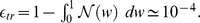


#### Fully connected network

**Figure 2:**
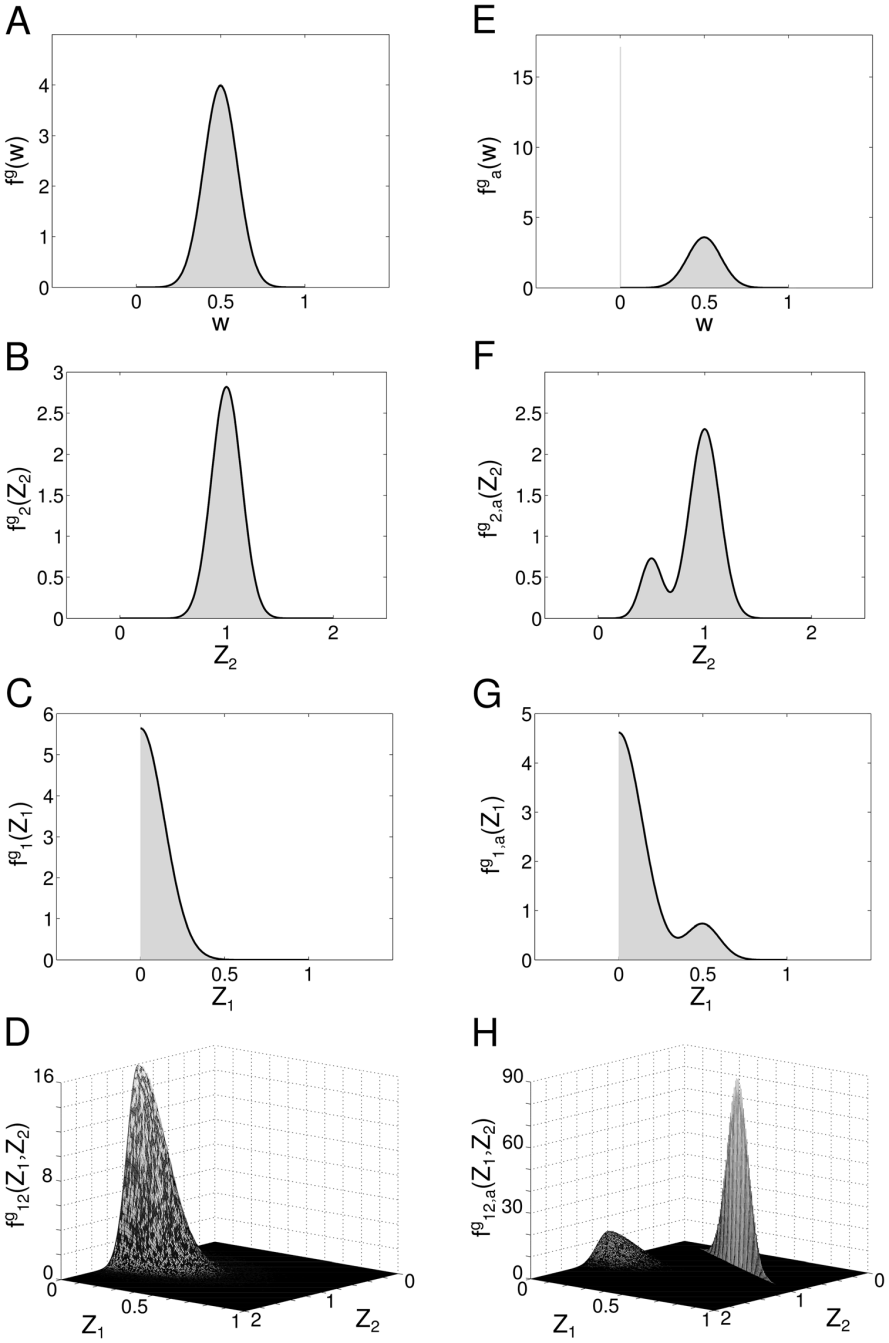
Probability density functions for the case of Gaussian-distributed connections. **A** Distribution of the Gaussian variable 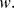 **B** Distribution of the sum 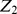 of two Gaussian-distributed variables. **C** Distribution of the absolute difference 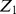 of two Gaussian-distributed variables. **D** Joint distribution of 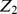 and 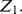 **E, F, G, H** The same as A, B, C and D but with pruning 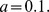 In all the figures, *Grey shaded area*: histograms from simulations, *Black lines and surfaces*: theoretical results (see Eq. (19)–(25)).

For the truncated Gaussian distribution defined above, the distribution of connections without pruning is (see also Fig. 2A):
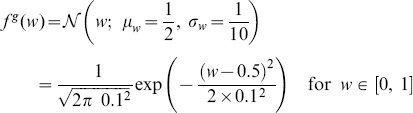
(18)where 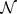 denotes the normal distribution. Since combinations of Gaussian distributions are also Gaussian distributions, we can immediately derive the PDF of 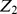 and 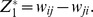 Then, 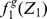 is simply the positive half of 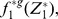 but scaled by a factor of two because of the normalization. We obtain (Fig. 2B,C):
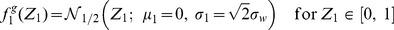
(19)

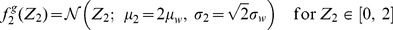
(20)where 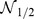 identifies the normalised (positive) half of a normal distribution. Similarly, the joint distribution 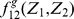 can be easily derived from the bivariate Gaussian of 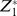 and 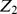 (Fig. 2D):
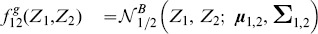
(21)with 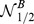 being the normalised half (where 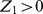) of a bivariate normal distribution.

#### Pruning

When taking pruning into account, each PDF can be considered as a mixture of the unpruned distribution and the contribution coming from the pruning. We can therefore write:
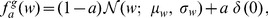
(22)

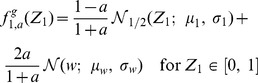
(23)

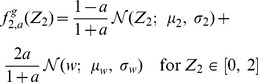
(24)


The above distributions are plotted in Fig. 2E, 2F, 2G.

Finally, the joint PDF is again a mixture model, with a univariate Gaussian peak profile on the line 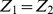 (Fig. 2H). Note that this peak can be described by the intersection of the plane 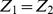 with the full unpruned bivariate normal distribution 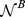 transformed to have its mean in 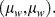 This operation implies a re-normalisation by 
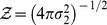
 of the resulting univariate Gaussian. Then, we can write:
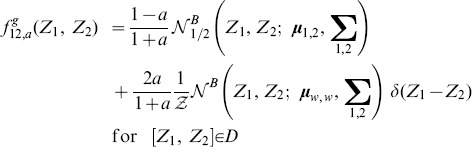
(25)


#### Correlation in the bivariate Gaussian

The correlation 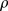 between 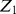 and 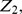 appearing in the off-diagonal terms of 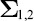, can be computed by running a numerical simulation. We estimated 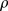 as a mean value over 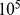 representations of a 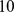-neuron network with random connections distributed according to 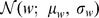 and with no pruning, i.e. 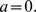 The result is 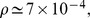 which allows to treat 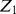 and 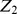 as independent variables and then to factorise the bivariate normal distribution Eq. (21) in the product of the two single distributions. Indeed, by introducing the Heaviside step function 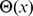 and the re-normalisation parameter 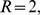 we can write:
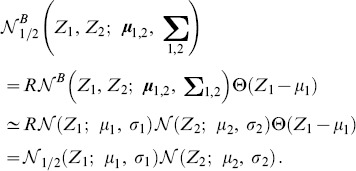
(26)


We note that the pruning case does not require a different calculation and can be treated as the 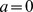 case. This is because we are describing the effect of the pruning with a separate (univariate) function, i.e. the halved bivariate normal distribution describes only the unpruned part of the network, see Eq. (25).

The suitability of this approximation is also certified by Fig. 2D,H, where the agreement between simulation results and theoretical fit with Eq. (26) is excellent.

#### Expected value and variance of 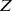


Now we can insert the expression of the joint distribution, Eq. (25), into Eq. (3) and Eq. (4):
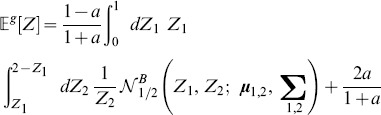
(27)

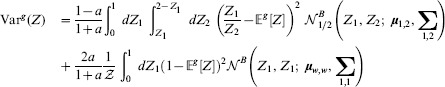
(28)


To calculate the above expression we use symbolic integration.

#### Expected value and variance of 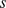


By plugging the above results into Eq. (3), (4), we obtain:
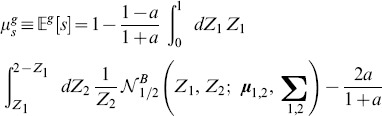
(29)

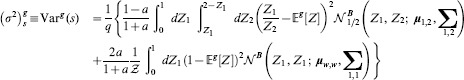
(30)


The four formulas Eq. (16), (17), (29), (30) are the final result of the statistical analysis and they will be discussed in the Results section.

### Model network with plastic weights

Below we describe the model neural network on which we will apply our symmetry measure.

#### Single-neuron dynamics

We simulated 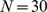 leaky integrate-and-fire neurons [27] with a firing threshold of 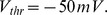 The sub-threshold dynamics of the electrical potential 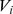 is given by:
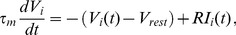
(31)where 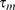 is the membrane time constant, 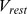 is the resting potential, 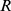 is the membrane resistance and 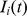 is the input signal. We chose 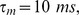 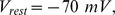 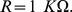 To introduce noise in the firing process of neurons, we implemented the escape noise model [28]. At each time-step 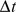 the probability that the neuron 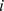 fires is given by:
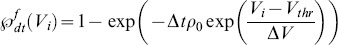
(32)where 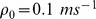 and 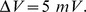 Once a neuron fires, its membrane potential is reset to the resting value.

#### Synaptic and External Inputs

The input 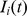 to each neuron has two components: a synaptic part, coming from the action potentials of the other neurons, and an external part, which is defined by the applied protocol:
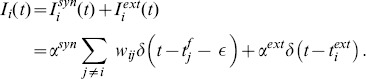
(33)


In the synaptic term, 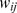 are the synaptic weights, 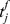 is the firing time of the presynaptic neuron 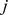 and 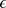 is a small positive number accounting for the delivering time of the electrical signal from the presynaptic to the postsynaptic neuron. The term 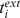 is the time course of the injected input, which is different from neuron to neuron and depends on the protocol we use (see Results section). Finally, the amplitudes 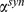 and 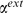 are fixed to the same value for all neurons. We chose 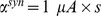 and 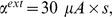 so that each external input forces the neurons to fire.

#### Plasticity

The efficacy of the synaptic connections is activity-dependent. Therefore, the unpruned elements of the adjacency matrix 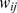 in Eq. (33) change in time by Spike-Timing Dependent Plasticity (STDP) mechanisms, i.e. passively driven by the input protocol and emerging internal dynamics, without the presence of a supervisory or reinforcement learning signal [29]–[31]. More specifically, we implemented the triplet STDP rule [18], [32], [33] with parameters from [32] (Visual cortex, nearest neighbour dataset), see Table 1, and we constrain the connections in 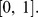 In this model, each neuron has two presynaptic variables 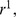 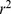 and two postsynaptic variables 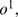 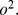 In the absence of any activity, these variables exponentially decay towards zero with different time constants:
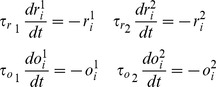
(34)whereas when the neuron elicits a spike they increase by 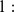


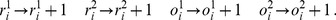
(35)


**Table 1:** List of parameters used for the case study.

Symbol	Description	Value
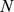	Number of neurons	30
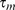	Membrane time constant	10 ms
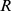	Membrane resistance	1 K 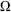
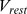	Resting and after-spike reset potential	−70 mV
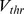	Threshold potential for spike emission	−50 mV
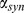	Voltage increase due to a presynaptic event	1 mV
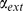	Voltage increase due to an external event	30 mV
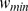	Lower bound for synaptic weights	0
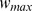	Higher bound for synaptic weights	1
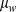	Mean value of Gaussian-distributed initial weights	0.5
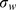	Variance of Gaussian-distributed initial weights	0.01
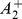	Amplitude of weights change - pair term in Long-Term Potentiation	4.6 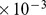
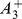	Amplitude of weights change - triplet term in Long-Term Potentiation	9.1 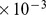
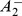	Amplitude of weights change - pair term in Long-Term Depression	3.0 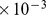
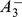	Amplitude of weights change - triplet term in Long-Term Depression	7.5 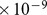
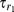	Decay constant of presynaptic indicator 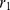	16.8 ms
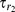	Decay constant of presynaptic indicator 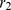	575 ms
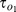	Decay constant of postsynaptic indicator 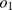	33.7 ms
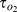	Decay constant of postsynaptic indicator 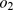	47 ms
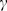	Learning rate for STDP	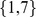
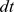	Discretisation time step	1 ms
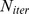	Number of independent repetitions of the experiment	50

STDP parameters are as in the nearest-spike triplet-model, described in [32].

Then, assuming that neuron 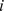 fires a spike, the STDP implementation of the triplet rule can be written as follows:
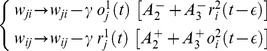
(36)where 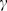 is the learning rate and 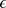 is an infinitesimal time constant to ensure that the values of 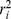 and 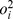 used are the ones right before the update due to the spike of neuron 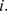 The learning rate used is 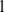 for the frequency protocol, 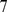 for the sequential protocol (see Results).

#### Reproducibility of results

All simulations were performed in MATLAB (The Mathworks, Natick, USA). Code is available from ModelDB [34], accession number: 151692.

## Results

We recall the definition of the symmetry measure 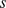 (Eq. 1):
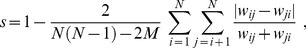
(37)where 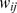 is the positive synaptic connection from neuron 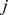 to neuron 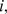 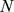 is the total number of neurons and 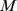 is the number of instances where both 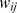 and 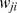 are zero, i.e. there is no connection between two neurons. The term 
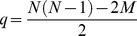
 is a normalisation factor that represents the total number of synaptic connection pairs that have at least one non-zero connection.

By using this definition, we were able to estimate the expected value and the variance of 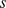 on random matrices (uniform and truncated Gaussian), see Eq. (16)–(17) and (29)–(30) correspondingly. This provides us a tool to estimate the significance of the “symmetry” or “asymmetry” of the adjacency matrix of a given network, shaped by learning, given the initial distribution of the synaptic connections prior to the learning process. The statistical analysis is particularly useful in cases where the developed configuration is not “clear-cut”, i.e. all connections have been turned to either bidirectional or unidirectional resulting in a symmetry measure almost 1 or 0, which is probably an artificial scenario, but rather in the intermediate cases, where we need a measure of how far away the value of the symmetry measure of a specific configuration is from that of a random configuration. Though here we focused on two specific random distributions, our methodology is applicable to other distribution choices.

### Hypothesis test

Having calculated the mean and variance of the symmetry measure 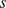 over random networks of a specific connectivity distribution, we are now able to directly evaluate the symmetry measure 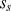 of a specific connectivity structure and conclude whether the symmetric or asymmetric structure observed is due to chance or it is indeed significant. A simple test is, for instance, to calculate how many standard deviations 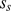 is away from 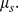 Equivalently, we can form the hypothesis that the configuration 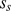 is non-random and calculate the *p*-value by:
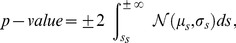
(38)where we implicitly assume that the distribution of the symmetry measure 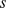 over all random networks is Gaussian. We can compare this result with the significance level we fixed, typically 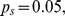 and we can then conclude the nature of the symmetry of the network with a confidence level equal to 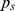 or reject the hypothesis.

### Pruning biases the network towards asymmetry

**Figure 3:**
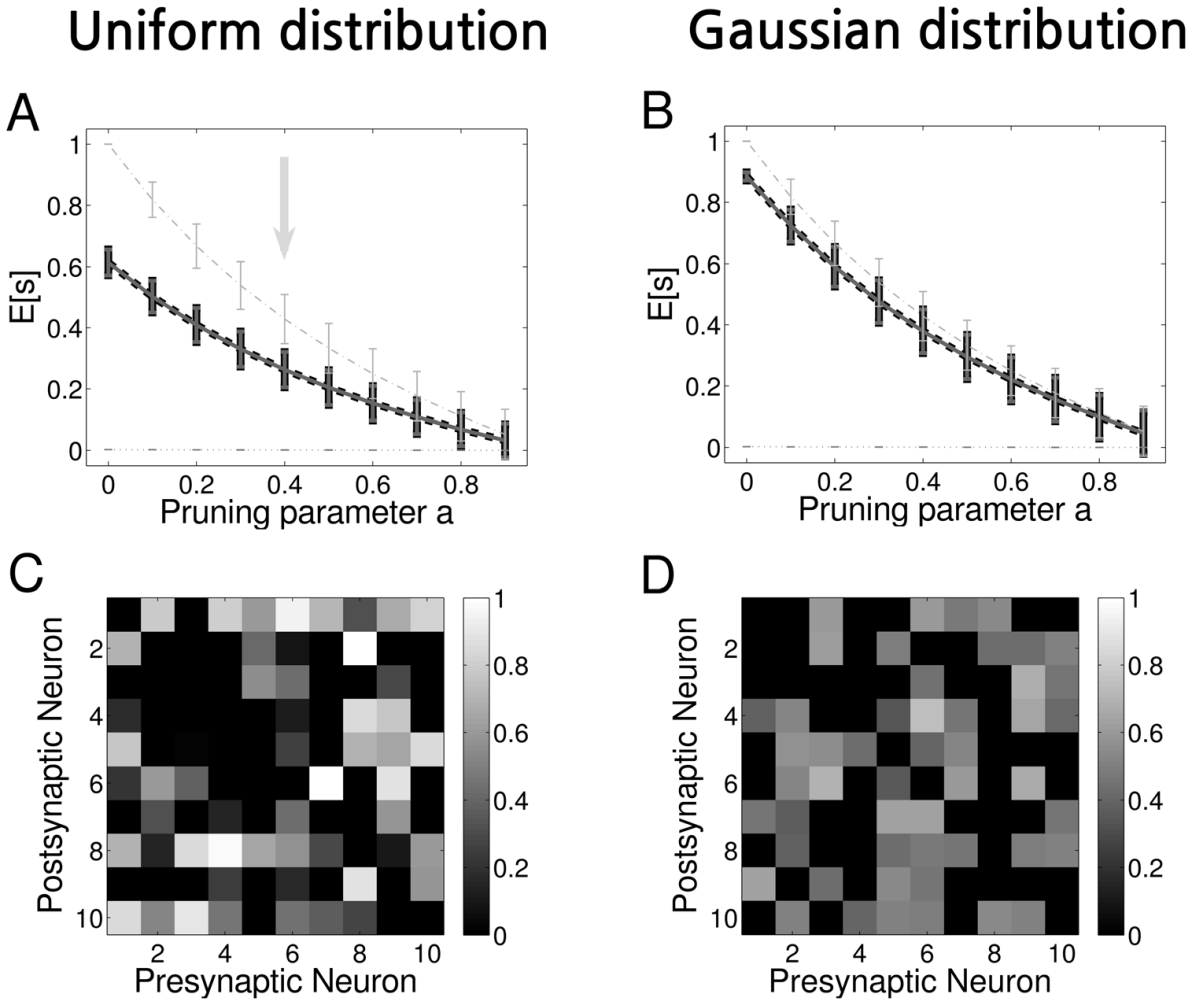
Final statistics of the symmetry measure. **A** Expected value and standard deviation of the symmetry measure as a function of the pruning for different types of networks with uniform weights distribution. The total length of each bar is two times the standard deviation. *Dashed light grey line*: simulations for symmetric networks, *Dash dotted light grey line*: simulations for asymmetric networks, *Solid dark grey line*: simulations for random networks, *Dashed black line*: theoretical results for random networks. **B** The same as A but with Gaussian-distributed random weights. **C** Example of an adjacency matrix in a particular random network with uniform weights distribution and pruning parameter 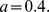 For this example 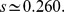 **D** The same as C but with Gaussian-distributed random weights. For this example 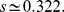

To demonstrate the validity of our analytical results, we compare them to simulation results. We generated a sample of 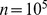 networks with 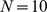 neurons with random connections with synaptic efficacies varying from 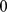 to 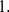 We evaluated the symmetry measure on each network by applying directly the definition of Eq. (37), and then we computed the mean value and variance of that sample. This process was repeated ten times, each one for a different value of the pruning parameter, 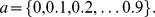 The final results are shown in Fig. 3A,B, together with the analytical results, see Eq. (16) and Eq. (29). Since numerical and analytical results overlap, we used a thicker (black) line for the latter. The agreement between theoretical findings, listed in Table 2, and numerical evaluations is excellent.

**Table 2:** Mean value and standard deviation of the symmetry measure as obtained from the theoretical analysis.

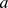	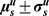	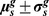
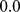	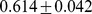	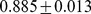
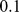	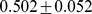	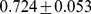
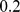	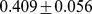	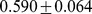
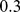	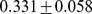	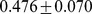
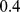	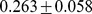	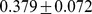
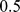	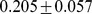	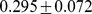
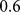	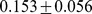	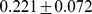
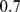	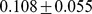	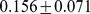
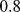	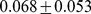	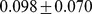
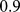	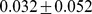	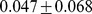

*Column 1.* Value of the pruning parameter 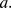 *Column 2.* Uniform distribution. *Column 3.* Gaussian distribution. These values are obtained with 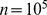 random networks of 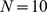 neurons and are plotted in Fig. 3.

We also considered two extreme cases, symmetric and asymmetric random networks, which respectively represent the upper and lower bound for the symmetry measure defined in Eq. (37). Symmetric random networks have been generated as follows: we filled the upper triangular part of the 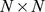 weights matrix with random values from the uniform/Gaussian distribution. We then mirrored the elements around the diagonal so as to have 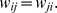 In the asymmetric case, instead, we generated a random adjacency matrix with values in 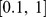 for the upper triangular part and in 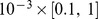 for the lower triangular part, so as to have 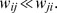 Then, we shuffled the adjacency matrix.

In Fig. 3A,B we contrast our results on random networks with numerical simulations of symmetric and asymmetric random networks: the dashed, light grey line (top line) shows the upper extreme case of a symmetric random network 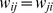 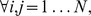 whereas the dash-dotted, light grey line (bottom line) shows the lower extreme case of a asymmetric random network 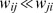 for 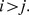


When we introduce pruning, the lower bound of 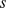 remains unchanged, whereas the more we prune the more a symmetric network appears as asymmetric.

### Gaussian-distributed synaptic efficacies bias the network towards symmetry

In Fig. 3C,D, we show the adjacency matrix 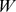 for a random pruned network with pruning parameter 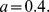 A network with uniformly distributed initial connectivity is shown in Fig. 3C and a network with Gaussian-distributed initial connectivity is shown in Fig. 3D. Black areas represent zero connection, 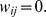 The “Gaussian” network has most of the connections close to the mean value 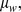 resulting in higher values for the symmetry measure than in the case of a uniform distribution, compare Fig. 3B with Fig. 3A.

**Figure 4:**
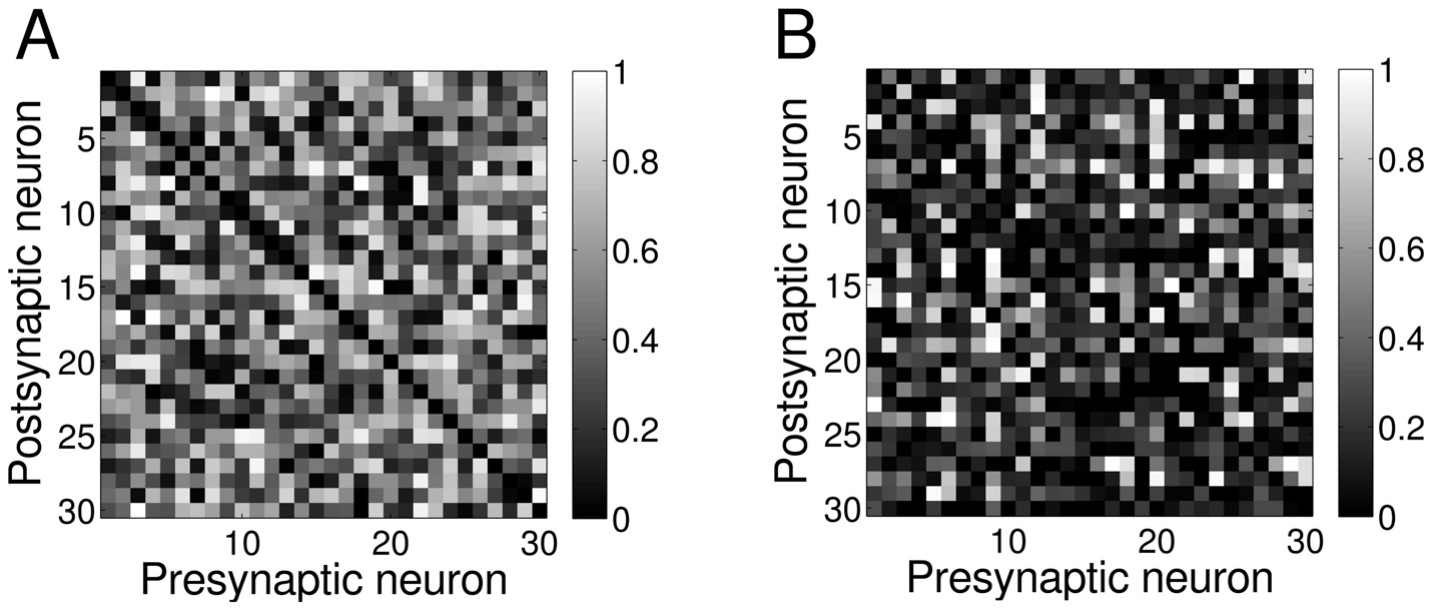
Symmetry and asymmetry depends on the distribution of the initial connectivity. **A** Example of an adjacency matrix in a random network with pruning parameter 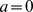 and symmetry measure 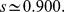 According with the *p*-value test with the null hypothesis of random connectivity and with a level of confidence of 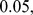 the symmetry of this network is significant if the distribution of the initial connections is uniform but is non-significant if the initial distribution of the connections is Gaussian. Therefore, in the first case it should be regarded as a non-random network whereas in the second case as a random network. **B** The same as A but with pruning parameter 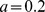 and symmetry measure 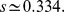 In this case, with the same hypothesis test, the situation is reversed: the network should be considered random for initial uniform distribution of connections, but non-random for initial Gaussian-distributed connections (see the discussion in the text).

This difference in the mean values of 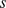 depending on the shape of the distribution implies that for example a weight configuration that would be classified as non-random under the hypothesis that the initial connectivity, before learning, is uniform, is classified as random under the hypothesis that the initial distribution of the connections is Gaussian. To more emphasise this point, we show in Fig. 4 the adjacency matrix of two different networks of 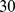 neurons. The first network, Fig. 4A, is a non-pruned network with 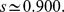 According with the values obtained from the statistical analysis (Table 2), if we assume that the connections of this network are randomly drawn from a uniform distribution, the *p*-value test (Eq. (38)) gives us *p*-value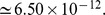 With the usual confidence level of 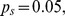 this is a significant result, implying that the network configuration is unlikely to be random. On the other hand, if we assume that the initial connectivity is drawn from a Gaussian distribution, we obtain *p*-value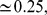 meaning that the network configuration should be considered random.

In Fig. 4B, we show a pruned network with 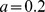 and 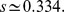 In this case the opposite is true: under the hypothesis of uniform random initial connectivity, the network should be considered random, as *p*-value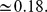 Under the hypothesis of Gaussian-distributed random initial connectivity, the network should be considered asymmetric, as *p*-value
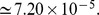


### Relation between symmetry measure and motifs

In what follows, we demonstrate the relation between our symmetry measure and unidirectional and bidirectional motifs. From the definition 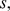 Eq. 37, we can deduct that in the extreme case of a network with unidirectional motifs, i.e. pairs of the form (0, x), 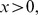 the symmetry measure will result in 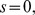 while in the case of bidirectional motifs i.e. pairs of the form (x, x), the symmetry measure will result in 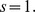 By inverting Eq. 3, we can derive the mean value for connection pairs 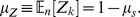 We can use now this value to define connection pairs in a network as unidirectional or bidirectional: if 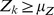 than 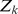 is a unidirectional motif, otherwise it is a bidirectional motif. In this way we relate unidirectional and bidirectional motifs to what is traditionally called single edge motif and second-order reciprocal motif, respectively. It is then expected that when 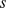 increases, the fraction of bidirectional motifs increases towards 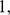 whereas the percentage of unidirectional motifs decreases towards 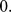


**Figure 5:**
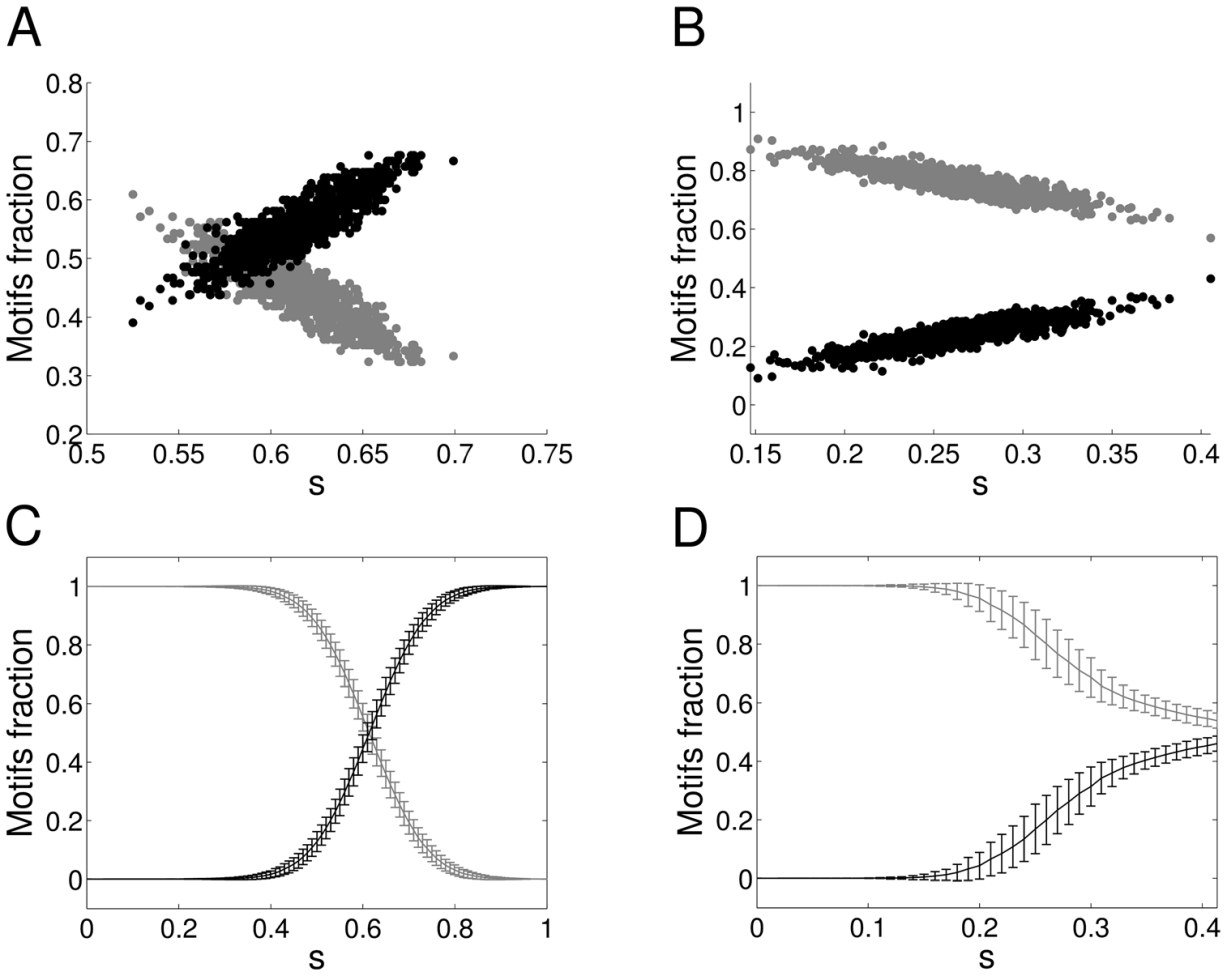
Symmetry measure reflects motifs formation. **A** Scatter plot of fraction of unidirectional and bidirectional motifs as a function of the symmetry measure for 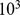 networks with uniform random connections and 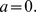 *Black dots*: bidirectional motifs, *Grey dots*: unidirectional motifs. For this typology 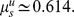 **B** The same as A but with pruning parameter 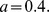 In this case 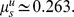 **C** Mean value and standard deviation (each bar is twice the standard deviation) of fraction of unidirectional and bidirectional motifs as a function of the symmetry measure for 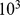 networks with half of the connections uniformly distributed and 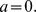 The second half of the connections were derived from the values of connection pairs 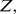 drawn from a Gaussian distribution with mean 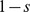 and standard deviation 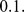 *Black line*: bidirectional motifs, *Grey line*: unidirectional motifs. **D** The same as C but with pruning parameter 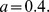

We show this relation in simulations by generating 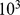 networks of 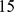 neurons each, with uniformly distributed random connections in 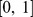 and no pruning. In this case the mean value of the symmetry measure is 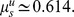 Using Eq. 3, we have 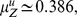 which is the value used to decide whether a connection pair is unidirectional or bidirectional. For each of these networks, we calculated the value of the symmetry measure and the fraction of unidirectional and bidirectional motifs and we plotted the results in Fig. 5A as a scatter plot (black circles - bidirectional motifs, grey circles - unidirectional motifs). Also, un Fig. 5B we show the analogous results obtained when we prune the connections with 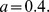 In both cases, a linear relation between 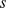 and motifs is evident.

Note that in both figures the restricted domain on the *s*-axis: this is determined by the range of 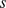 values that correspond to random networks. If we want to extend this range, we need to consider networks that are not random any more. We achieve this by fixing a distribution for connection pairs 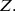 Once we decide on the desirable value of 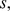 in our case the whole zero to one spectrum, we can use a distribution (e.g. Gaussian) with mean 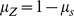 and a chosen variance to draw the values of all the connection pairs in the network. Following this procedure, we fill the upper triangular part of the 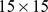 weights matrix with random values from the uniform/Gaussian distribution, and derive the other half of the weights by inverting the definition of 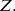 As a PDF(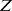) we chose a Gaussian distribution around 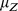 with 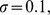 except for the extreme cases (near 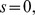 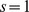) where 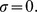 With this technique of creating networks, we sampled the entire domain of 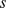 in steps of 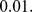 For each value, we again generated 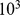 networks of 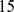 neurons with (half of the) weights uniformly distributed, and then we computed the mean value and standard deviation. Results are shown in Fig. 5C,D respectively for unpruned and pruned (with 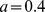) networks (black line - bidirectional motifs, grey line - unidirectional motifs). We can see that Fig. 5C,D correctly reproduce the linear regime observed in Fig. 5A,B for values of 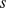 close enough to 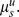


Due to the method by which we generated networks, the shape of the distribution of half of the weights does not affect the shape of the dependence in Fig. 5C,D. Indeed, if we choose half of the connections to be Gaussian-distributed, we will observe only a shift in both curves as they have to cross at 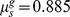 (results not shown).

### Symmetry measure and eigenvalues

In the definition of our symmetry measure we have deliberately excluded 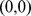 connection pairs. This was a conscious decision for mathematical and practical reasons, see Methods. As a consequence, pairs of the form 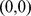 do not contribute to the evaluation of the symmetry of the network. Instead, pairs of the form 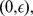 with 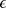 very small, contribute to the asymmetry of the network according to our specific choice of symmetry measure (leading to 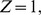 see Methods). Here we further motivate this choice via a comparison of our measure to the evaluation of the symmetry via the matrix eigenvalues, for three types of networks: (i) symmetric, where each connection pair consists of synapses of the same value, (ii) asymmetric, where every connection pair has one connection set to a small value 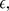 and (iii) random, where connections are uniformly distributed. We demonstrate that our measure has a clear advantage over the eigenvalues method, in particular when pruning is introduced. This difference in performance lays in the different ways that 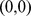 and 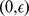 are treated by our measure.

**Figure 6:**
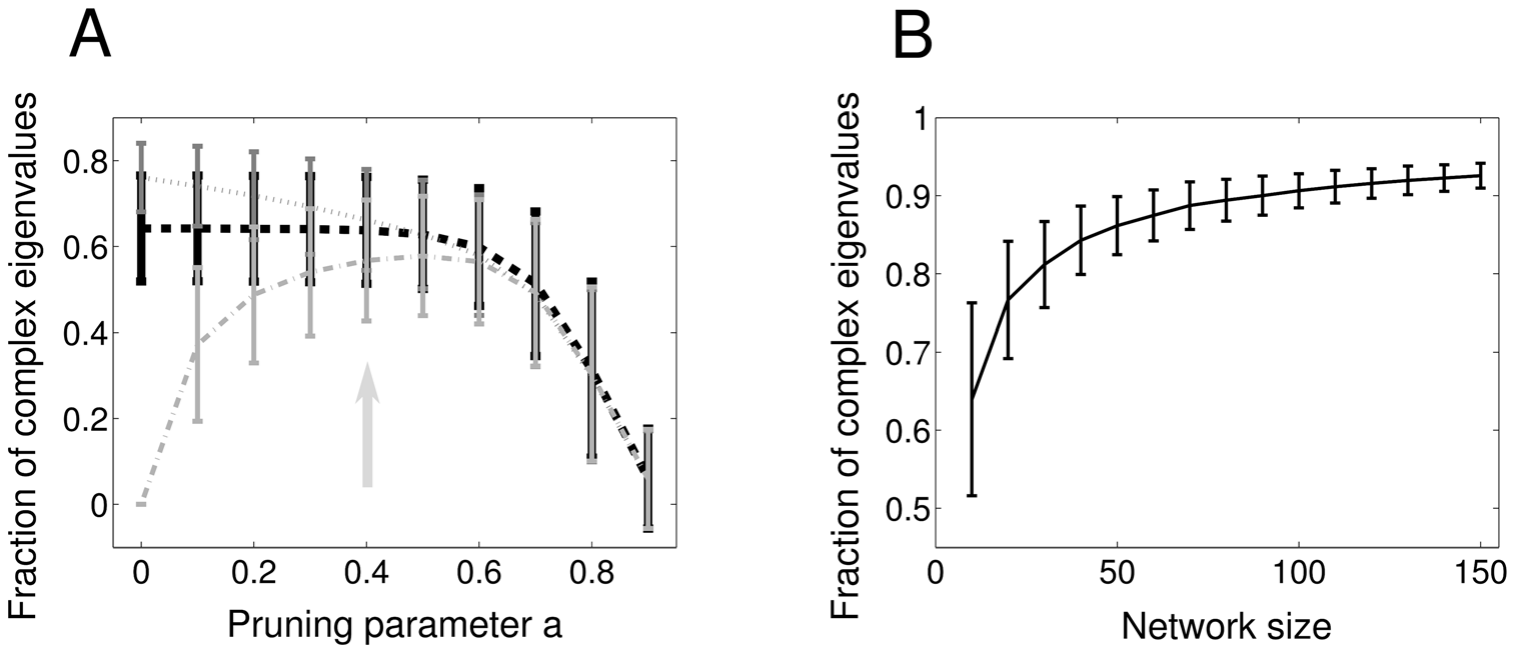
Eigenvalues and network structure. **A** Expected value and standard deviation of the fraction of complex eigenvalues as a function of the pruning for different types of networks of 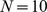 neurons with uniform weight distribution. The total length of each bar is two times the standard deviation. *Dotted, dark grey line*: simulations for asymmetric networks, *Dashed, black line*: simulations for random networks, *Dash-dotted, light grey line*: simulations for symmetric networks. **B** Fraction of complex eigenvalues as a function of network size for random networks with uniform weights distribution. Pruning parameter 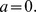

A crucial property of the real symmetric matrices is that all their eigenvalues are real. Fig. 6A depicts the fraction of complex eigenvalues vs the pruning parameter 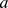 for a symmetric (dash-dotted, light grey line) asymmetric (dotted, dark grey line) and random (dashed, black line) matrix with uniformly distributed values, similar to Fig. 3A, with the same statistics (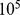 networks of 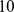 neurons). As expected, if no pruning takes place (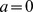), symmetric matrices have no complex eigenvalues and are clearly distinguishable from random and asymmetric matrices. On the contrary, both random and asymmetric matrices have a non-zero number of complex eigenvalues, which increases with a higher degree of asymmetry, leading to a considerable overlap between these two cases, differently from what happens with our measure in Fig. 3A.

As we introduce pruning, the mean of the complex eigenvalues of the three distinctive types of network moves towards the same value, an increase for the symmetric network and decrease for the random and non-symmetric networks. This is expected as pruning specific elements will make the symmetric network more asymmetric while it will increase the symmetry of the asymmetric network by introducing pairs of the form 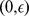 or 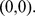 The 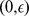 pairs are due to the construction of the asymmetric network, where half of the connections are stochastically set to very low values. This continues till 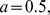 after which further pruning reduces the number of complex eigenvalues of all networks: a high level of pruning implies the formation of more 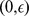 or 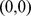 pairs for the asymmetric network and more 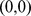 pairs for the symmetric network. In Fig. 6B we show the dependence of the fraction of complex eigenvalues for uniform random matrices on their size.

Comparing Fig. 6A to Fig. 3A, we observe that our symmetry measure offers excellent discrimination between the symmetric, asymmetric and random matrices for e.g. 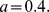 This is despite the fact that the structure of the asymmetric matrix *per se* has become less asymmetric and the structure of the symmetric matrix has become more asymmetric due to the pruning, as it is confirmed by the overlapping fraction of complex eigenvalues for asymmetric and random matrices (Fig. 6A). In our measure 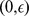 pairs are treated as asymmetric, 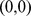 pairs are ignored, and the bias that pruning introduces is taken into account allowing for good discrimination for all types of matrices, even beyond 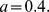


### Case study: Monitoring the connectivity evolution in neural networks

We demonstrate the application of the symmetry measure to a network of neurons evolving in time according to a Spike-Timing Dependent Plasticity (STDP) “triplet rule” [32] by adopting the protocols of [18]. These protocols are designed to evolve a network with connections modified according to the “triplet rule”, to either a unidirectional configuration or bidirectional configuration, with the weights being stable under the presence of hard bounds. We have deliberately chosen a small size network as a “toy-model” that will allow for visual inspection and characterisation at the mesoscopic scale.

**Figure 7:**
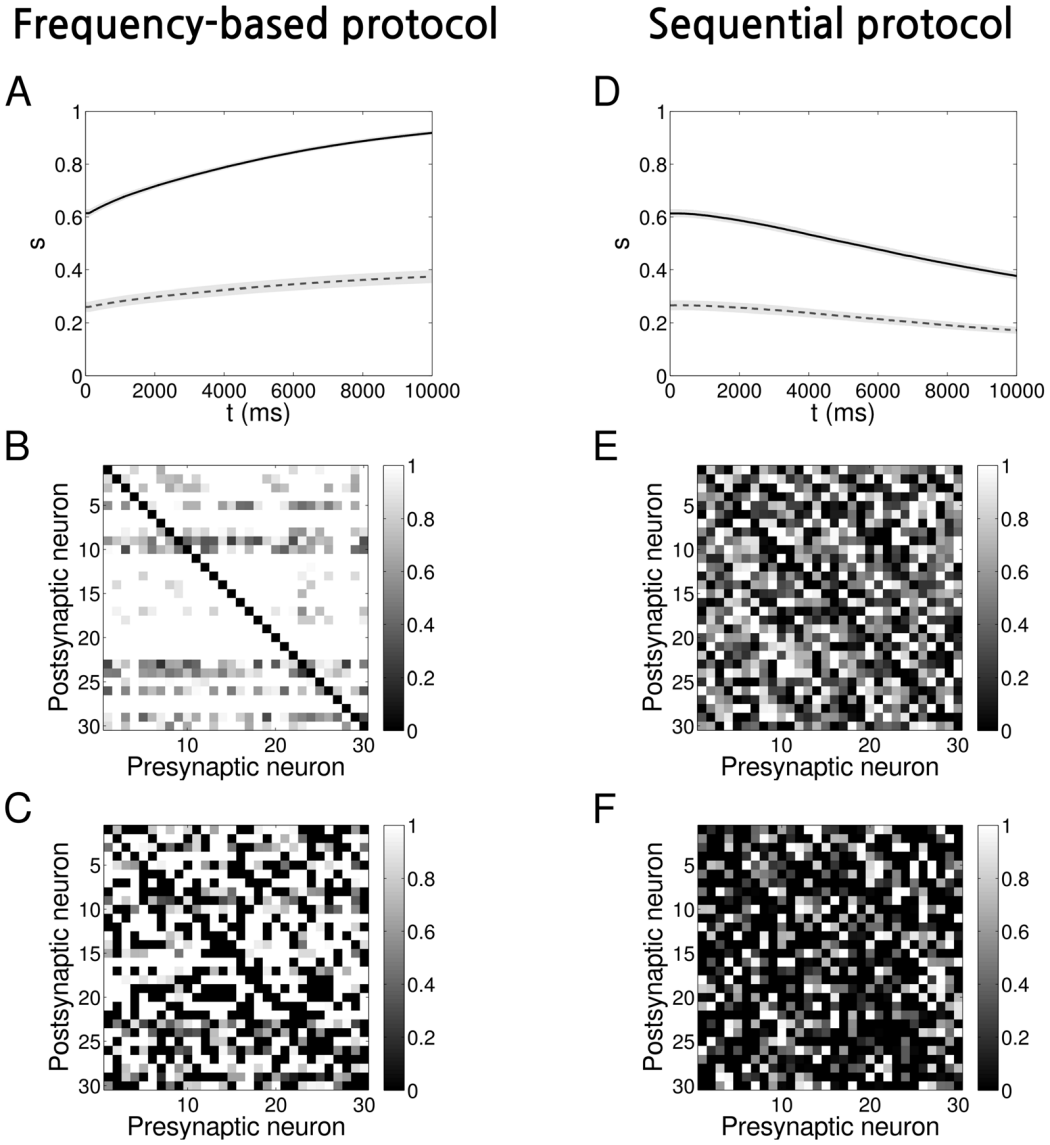
Evolution of networks with STDP and initially uniform weights distribution. **A** Time evolution of the symmetry measure when a frequency protocol is applied on a network, shown as average over 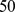 representations. The shaded light grey areas represent the standard deviation (the total length of height of each band is twice the standard deviation). *Solid black line*: no pruning, *Dashed grey line*: with pruning 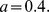 **B** Example of an adjacency matrix at the end of the learning process for a network with the frequency protocol and no pruning. For this example 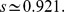 **C** The same as B but with pruning 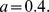 For this example 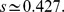 **D, E, F** The same as A, B and C but with the sequential protocol applied. The connectivity matrix in panel E has 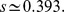 The connectivity matrix in panel F has 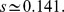

**Figure 8:**
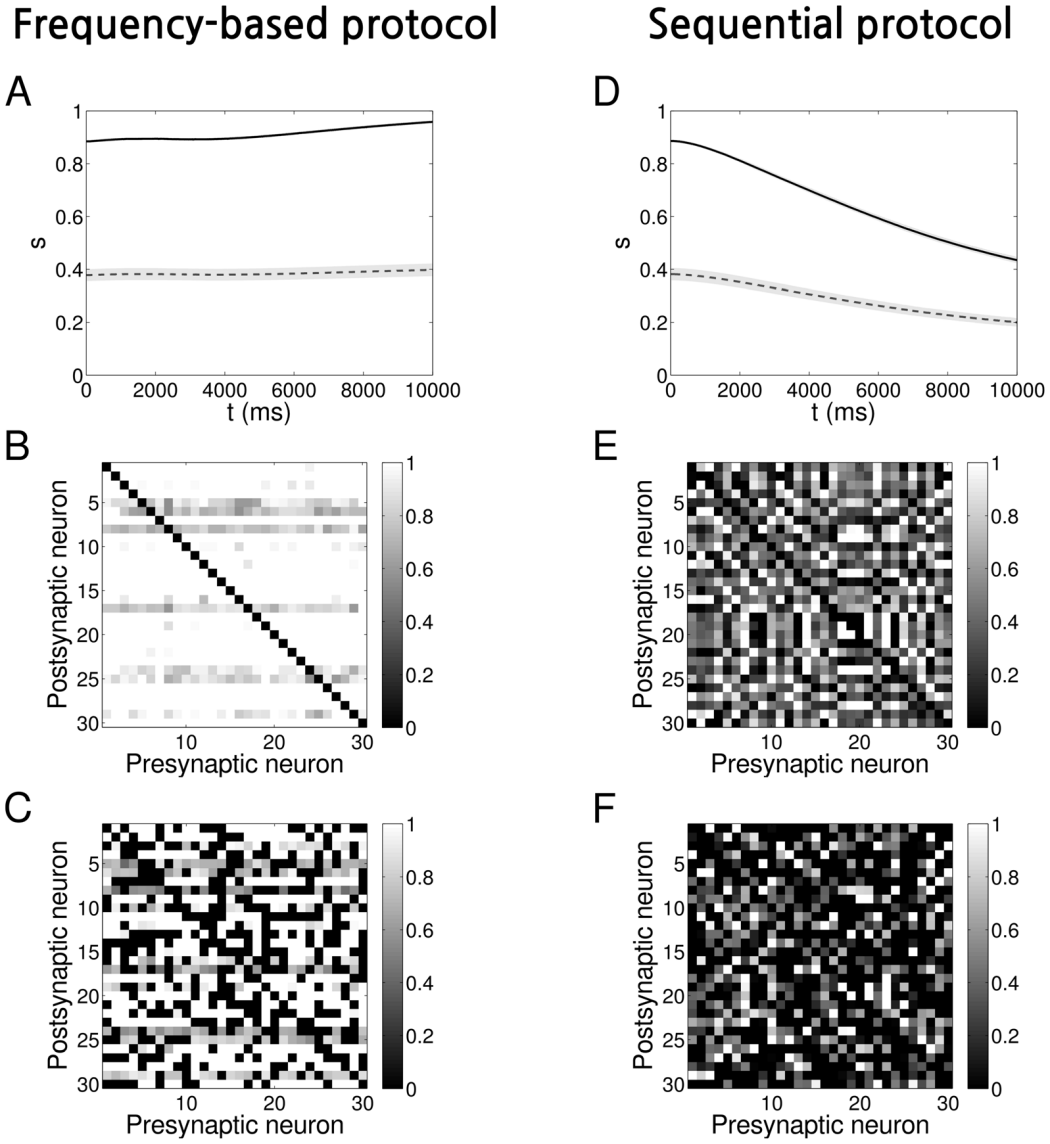
Evolution of networks with STDP and initially Gaussian-distributed weights. **A** Time evolution of the symmetry measure when a frequency protocol is applied on a network, shown as average over 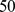 representations. The shaded light grey areas represent the standard deviation (the total length of height of each band is twice the standard deviation). *Solid black line*: no pruning, *Dashed grey line*: with pruning 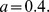 **B** Example of an adjacency matrix at the end of the evolution for a network with frequency protocol and no pruning. For this example 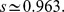 **C** The same as B but with pruning 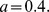 For this example 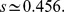 **D, E, F** The same as A, B and C but with the sequential protocol applied. The connectivity matrix in panel E has 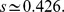 The connectivity matrix in panel F has 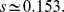

We simulated 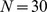 integrate-and-fire neurons (see Methods section for simulation details) initially connected with random weights 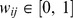 drawn from either a uniform (Fig. 7) or a Gaussian (Fig. 8) distribution (see Table 1 for parameters). Where a pruning parameter is mentioned, the pruning took place prior to the learning procedure: with a fixed probability some connections were set to zero and were not allowed to grow during the simulation.

Our choice allows us to produce an asymmetric or a symmetric network depending on the external stimulation protocol applied to the network. Since the amplitude of the external stimulation we chose (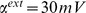) is large enough to make a neuron fire every time it is presented with an input, the firing pattern of neurons reflects the input pattern and we can indifferently refer to one or another. The asymmetric network has been obtained by using a “sequential protocol”, in which neurons fire with the same frequency in a precise order one after the other, with 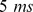 delay, see also [18]. The symmetric network is produced by applying a “frequency protocol”, in which each neuron fires with a different frequency from the values 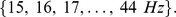 In both cases, the input signals were jittered in time randomly with zero mean and standard deviation equal to 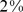 of the period of the input itself for the frequency protocol, to 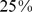 of the delay for the sequential protocol. Depending on the protocol, we expect the neurons to form mostly unidirectional or bidirectional connections during the evolution.

The time evolution for both protocols and initial distributions is shown in Figs 7A,D (uniform) and 8A,D (Gaussian). Each panel represents the evolution of the symmetry measure averaged over 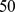 different representations for both fully connected networks (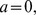 solid black line) and pruned networks (e.g. 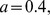 dashed grey line). The shaded area represents the standard deviation. The time course of the symmetry measure can be better understood with the help of the Fig. 3. At the beginning, the values of 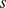 reflect what we expect from a random network. Afterwards, as the time passes, the learning process leads to the evolution of the connectivity. As expected, the frequency protocol induces the formation of mostly bidirectional connections, leading to the saturation of 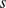 towards its maximum value, depending on the degree of pruning. On the other hand, when we apply the sequential protocol, connection pairs develop a high degree of asymmetry, the values of 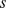 decreasing towards its minimum. Connections were constrained to remain inside the interval 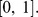


The final connectivity pattern can be inspected by plotting the adjacency matrix 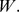 In Fig. 7B,C and 8B,C we give an example of 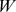 at the end of the evolution for one particular instance of the 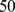 networks when the frequency protocol is applied. Similarly, in Fig. 7E,F and 8E,F we show the results for the sequential protocol. The corresponding values of 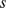 for each of the examples in the figures are listed in Table 3. In the case that 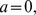 a careful inspection of Fig. 7B, 8B indicates that connectivity is bidirectional: all-to-all strong connections have been formed. On the other hand, In Fig. 7E, 8E, trying to determine if there is a particular connectivity emerging in the network starts to be considerably tough. However, by using our symmetry measure (see values in Table 3) we can infer that the connectivity is unidirectional. In the pruned networks, however, see Fig. 7C, 8C and Fig. 7F, 8F, the formation of bidirectional and unidirectional connection pairs is not as obvious as for 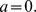 We therefore refer again to the Table 3 and compare the values of 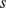 with 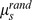 and with 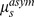 or 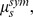 depending on the case. We can then verify that the learning process has significantly changed the network and its inner connections from the initial random state.

**Table 3:** Symmetry measure and *p*-value for different types of network.

Type	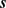	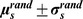	*p*-value	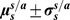
UF 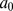	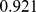	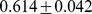	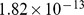	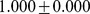
UF 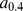	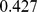	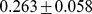	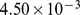	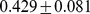
US 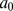	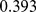	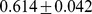	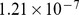	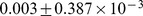
US 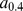	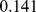	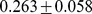	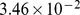	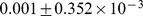
GF 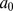	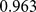	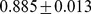	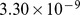	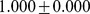
GF 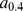	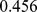	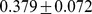	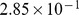	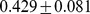
GS 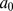	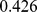	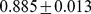	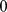	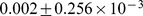
GS 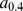	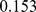	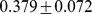	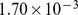	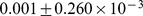

*Column 1.* Network type. 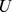 = Uniform distribution, 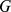 = Gaussian distribution, 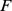 = Frequency protocol, 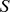 = sequential protocol, 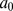 = No prune, 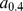 = pruning of 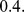 *Column 2.* Value of the symmetry measure for one instance of each type. *Column 3.* Results from the previous statistical analysis on random networks. *Column 4.* Corresponding *p*-value from Eq. 38. *Column 5.* Results from the previous statistical analysis for the corresponding closest extreme case – symmetric network for frequency protocol and asymmetric network for sequential protocol. 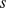 means symmetric and 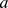 asymmetric.

We can rigorously verify the above conclusions via a statistical hypothesis test such as the *p*-value test, which in essence quantifies how far away the value of our symmetry measure 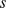 of our final configuration is from the initial, random configuration (see also Methods). In Table 3 we show the *p*-values corresponding to the null hypothesis of random connectivity for the examples in the Fig. 7, 8. Once we set the significance level at 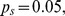 we can verify that, except for the case of pruned network with initially Gaussian-distributed connections where a frequency protocol has been applied (i.e. GF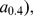 the *p*-values are significant, implying the rejection of the null hypothesis. This is also justified by Fig. 3A, B: when we increase the pruning, the mean value of the symmetry measure of the fully symmetric network approaches that of the pruned random network and in particular for the case where the weight are randomly Gaussian-distributed.

## Summary

The study of the human brain reveals that neurons sharing the same cognitive functions or coding tend to form clusters, which appear to be characterised by the formation of specific connectivity patterns, called motifs. We, therefore, introduced a mathematical tool, a symmetry measure 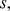 which computes the mean value of the connection pairs in a network, and allows us to monitor the evolution of the network structure due to the synaptic dynamics. In this context, we applied it to a number of evolving networks with plastic connections that are modified according a learning rule. After the network connectivity reaches a steady state as a consequence of the learning process, connectivity patterns develop. The use of the symmetry measure together with the statistical analysis and the *p*-value test allow us both to quantify the connectivity structure of the network, which has changed due to the learning process, and observe its development. It also allows for some interesting observations. (i) Introducing a fixed amount of pruning in the network prior to the learning process biases the adjacency matrix towards an asymmetric configuration. (ii) A network configuration that appears to be symmetric under the assumption of a uniform initial distribution is random under the assumption of a Gaussian initial distribution.

Statements on non-random connectivity in motifs experimental work, e.g. [14], [20] are supported by calculating the probability of connectivity in a random network and then distributing it uniformly: this becomes the null hypothesis. This was a most suitable approach given the paucity of data. If, however, the null hypothesis consisted of a Gaussian-distributed connectivity, then a higher number of bidirectional connections would be expected, as suggested by our analysis.

It is also possible that in a large network, learning processes are only modifying a subset of the connections, forming motifs that might be unobserved if the symmetry measure is applied to the whole adjacency matrix. In such cases, algorithms of detecting potential symmetric or asymmetric clusters would detect the area of interest and the symmetry measure presented here reveals the evolution of the structure and its significance.
